# Restricted diversity of dental calculus methanogens over five centuries, France

**DOI:** 10.1038/srep25775

**Published:** 2016-05-11

**Authors:** Hong T. T. Huynh, Vanessa D. Nkamga, Michel Signoli, Stéfan Tzortzis, Romuald Pinguet, Gilles Audoly, Gérard Aboudharam, Michel Drancourt

**Affiliations:** 1 Aix Marseille Université, Faculté d’Odontologie, Marseille 13005, France; 2 Aix Marseille Université, URMITE, UMR CNRS 7278, IRD 198, INSERM 1095, Faculté de Médecine, Marseille 13005, France; 3 Aix-Marseille Université, UMR 7268 ADES, EFS CNRS, Faculté de Médecine Secteur Nord, Marseille, France; 4 Institut National de Recherches Archéologiques Préventives, Paris, France

## Abstract

Methanogens are acknowledged archaeal members of modern dental calculus microbiota and dental pathogen complexes. Their repertoire in ancient dental calculus is poorly known. We therefore investigated archaea in one hundred dental calculus specimens collected from individuals recovered from six archaeological sites in France dated from the 14^th^ to 19^th^ centuries AD. Dental calculus was demonstrated by macroscopic and cone-beam observations. In 56 calculus specimens free of PCR inhibition, PCR sequencing identified *Candidatus* Methanobrevibacter sp. N13 in 44.6%, *Methanobrevibacter oralis* in 19.6%, a new *Methanomassiliicoccus luminyensis-*like methanogen in 12.5%, a *Candidatus* Nitrososphaera evergladensis-like in one and *Methanoculleus bourgensis* in one specimen, respectively. One *Candidatus* Methanobrevibacter sp. N13 dental calculus was further documented by fluorescent *in situ* hybridization. The prevalence of dental calculus *M. oralis* was significantly lower in past populations than in modern populations (P = 0.03, Chi-square test). This investigation revealed a previously unknown repertoire of archaea found in the oral cavity of past French populations as reflected in preserved dental calculus.

Dental plaque is thought to progressively build up, calcify and turn into dental calculus, mainly in individuals with poor daily dental hygiene and poor access to professional care^1^. Indeed, dental calculus cannot be removed with a toothbrush and only a dental professional can remove it during an oral cleaning. Importantly, microorganisms found in dental plaque in modern populations are also implicated in periodontitis^2^. Investigations of dental plaque in modern populations in Italy, Switzerland, USA, Japan, China, Germany, Brazil and France using culture and culture-independent investigations, found methanogens^3^,^4^,^5^,^6^,^7^,^8^,^9^. Accordingly, the methanogen *Methanobrevibacter oralis*, initially isolated from the subgingival plaque of healthy subjects, has subsequently been detected in periodontitis-related lesions and peri-implant pockets^2^,^4^,^10^. Several studies have further suggested that *M. oralis* is involved in periodontitis^2^ and we confirmed this linkage in a previous study in which we found that *M. oralis* load is significantly correlated with the severity of periodontitis^3^. Recently, we isolated a new methanogen *Candidatus* Methanobrevibacter sp. N13, along with *Methanobrevibacter smithii*, from periodontitis lesions in addition to *M. oralis*^11^. These data indicated that some methanogens found in the dental plaque, are implicated in dental disease.

Microorganisms stuck in dental calculus in past populations were investigated in order to help understand oral hygiene in the past^12^,^13^. No previous studies, however, have focused on methanogens in ancient dental calculus and their presence in such specimens is only known through two metagenomic analyses of dental calculus dated between 7550–400 BP and c. 950–1200 CE. These metagenomic analyses revealed that archaea comprised 17.10^−6^ of total reads and that methanogens were clearly dominant, being accompanied by only a small percentage of halophilic euryarchaeota^14^,^15^.

We therefore specifically investigated methanogens in a sample of dental calculus collected from 100 individuals dated from the 14^th^ to the 19^th^ centuries in France.

Initially, we aimed to confirm the presence of dental calculus in the sample under investigation. Macroscopic and cone-beam observations revealed the presence of dental calculus in the cervical one-third of the tooth crown in all the studied teeth. Dental calculus was then removed from every tooth for further analyses.

In a second step, methanogen-specific sequences were tentatively detected in every dental calculus specimen by means of PCR sequencing. In each PCR run, extraction and PCR amplification-negative controls remained negative but detection of an internal control indicated that only 56 (56%) dental calculus specimens were free of PCR inhibition. Among these 56 dental calculus specimens, 42 (75%) were positive for methanogen DNA evidenced by a 16S rRNA gene sequence in 40 (71.4%) samples and by a *mcr*A sequence in 28 (50%) samples. The 16S rRNA gene sequence was determined directly from the dental calculus specimen in 37 specimens (N17A, 385, 153, D314, GenBank LN610761; N17G, 529, 502, 497, 339, 145, 140, D353, D264, D208, GenBank LN610762; N57, N58C, N79, GenBank LN610763; N58T, GenBank LN610764; N70, GenBank LN610765; N71, P2, P4, P14, P26, F6, D222, GenBank LN610766; F27, GenBank LN827537; P27, GenBank LN827540; 138, GenBank LN827541; 142, GenBank LN827542; 486, GenBank LN827543; N102, D238, D362, GenBank LK054637; 105, 123, 356, 363, D230, GenBank LK054634). As for three other dental calculus specimens, direct sequencing indicated mixed sequences and the 16S rRNA gene sequences were determined after sequencing 10 clones for each specimen (N89–12, GenBank LN827538 and N89–2, GenBank LN827539; P16, P22, GenBank LN827539). The *mcr*A gene was determined directly from the dental calculus in 28 specimens (N10, P4, GenBank LN624393; N17, GenBank LN624394; N57, N58, N87, 529, 123, 140, 502, 497, 363, 385, 339, 356, 145, 153, D314, D353, D264, D208, GenBank LN624395; N70, GenBank LN624396; N71, GenBank LN624397; P14, P26, GenBank LK054628; F6, D222, D238, GenBank DQ251045).

In a third step, sequence analyses identified *Candidatus* Methanobrevibacter sp. N13 in 23 (41.1%) specimens, *M. oralis* in 11 (19.6%) specimens and *Methanoculleus bourgensis* in one specimen (1.8%) (Table 1, Fig. 1), respectively. Further 16S rRNA gene cloning revealed a sequence exhibiting 86% similarity with *M. luminyensis* (ref|NR_118098.1|) further referred to herein as *M. luminyensis*-like, in calculus specimen P22; a mixed *M. luminyensis*-like and *Candidatus* Methanobrevibacter sp. N13 in calculus specimen P16 and a mixed *M. luminyensis*-like, *Candidatus* Methanobrevibacter sp. N13 and a sequence exhibiting 87% similarity with the soil inhabitant *Candidatus* Nitrososphaera evergladensis (CP007174.1) in calculus specimen N89. Phylogenetic analysis further indicated that calculus specimens P27, 138, 142 and 486 also contained three 350–550-bp *M. luminyensis*-like sequences. The observation of one single nucleotide polymorphism in *Candidatus* Methanobrevibacter sp. N13 16S rRNA gene sequence in calculus specimens N17 and N58 (site A) and in calculus specimen 502 (site E) suggested that two *Candidatus* Methanobrevibacter sp. N13 strains could infect the same dental calculus. Indeed, Methanobrevibacter sp. N13 genome contains only one copy of the 16S rRNA gene excluding any heterogeneity in the same organisms (M. Drancourt, unpublished data). Methanogens were later directly observed in one PCR-positive dental calculus sample (D353) from site F by blind fluorescence *in situ* hybridization (FISH), while PCR-negative dental calculus samples and the negative control samples remained negative (Fig. 2). This finding represents the first detection of ancient methanogens by FISH.

Altogether, *Candidatus* Methanobrevibacter sp. N13 was found in 25 of 56 (44.6%) calculus specimens obtained from five sites spanning four centuries, *M. oralis* was found in 11 of 56 (19.6%) calculus specimens from four sites spanning three centuries. *M. luminyensis*-like was found in seven of 56 (12.5%) calculus specimens from four sites spanning four centuries. A *Candidatus* Nitrososphaera evergladensis-like methanogen was found in one specimen at site A, while *M. bourgensis* was observed in one calculus specimen obtained from the 19^th^ century site C. Intriguingly, *M. bourgensis* had previously only been isolated from a sewage sludge digester (Fig. 3).

We then compared the molecular detection of *M. oralis* in ancient dental calculus samples with that of modern-day ones^3^. As we had no clinical evidence for periodontal disease in ancient individuals, these comparisons were done assuming that ancient populations exhibited either the same ratio of healthy/unhealthy individuals as modern population in France (28.2%; (scenario 1)^16^ or only unhealthy individuals (scenario 2) or only healthy individuals (scenario 3). These comparisons indicated that the prevalence of *M. oralis* was lower in ancient populations than in modern-day population^3^ (P = 0.06, Cramer’s V = 0.2; P = 0.0002, Cramer’s V = 0.37; P = 0.19, Cramer’s V = 0.12, respectively).

There is no molecular study of the prevalence of dental calculus methanogens other than *M. oralis*. However, a recent study indicated that living methanogens were isolated in culture in 46.3% dental calculus samples collected in French individuals^11^. In particular, *M. oralis* was isolated in 47.7% dental calculus samples and *Candidatus* Methanobrevibacter sp. N13 was in 4.6% dental calculus samples. *M. smithii* however, though previously isolated from dental plaque samples obtained from modern French populations and detected in past populations in Germany, Poland and England^14^,^15^, was not detected in the ancient calculus samples tested here. By contrast, *M. luminyensis*-like, *M. bourgensis* and a *Candidatus* Nitrososphaera evergladensis-like methanogen found in the historic French samples included in the current study have never been detected in modern populations (Fig. 3).

Thus, it appears that regardless of the method used for the detection of methanogens in the dental plaque, the prevalence and diversity of methanogens in dental calculus have decreased significantly over the course of the past seven centuries. The most obvious change is the replacement of *Candidatus* Methanobrevibacter sp. N13 by *M. oralis* and *M. smithii*: a change apparently specific to the 21^st^ century. Here, we studied dental calculus specimens from France only in order to avoid any potential bias due to geographical variation of the microbiota repertoire, as well established for the intestinal microbiota for example^17^. However, we had no anthropological or historical data for further rigorous interpretation of the microbiological data. Nevertheless, it is tempting to associate modifications in the methanogen dental calculus repertoire with changes in food. Some studies of ancient dental calculus have shown that carbohydrate-rich diets correlated with a sharp increase in the prevalence of cariogenic *Streptococcus mutans*^13^. At the opposite the “red-complex” including *Porphyromonas gingivalis, Treponema gingivalis* and *Tannerella forsythia* has been associated with periodontitis in Medieval populations as it is in modern-day populations despite changes in diet^15^.

Understanding these factors is of interest as methanogens, and chiefly *M. oralis*, have been implicated in periodontitis, a disease with major nutritional consequences in certain populations such as elderly people^18^.

## Methods

### Archeological samples

**Figure 3:**
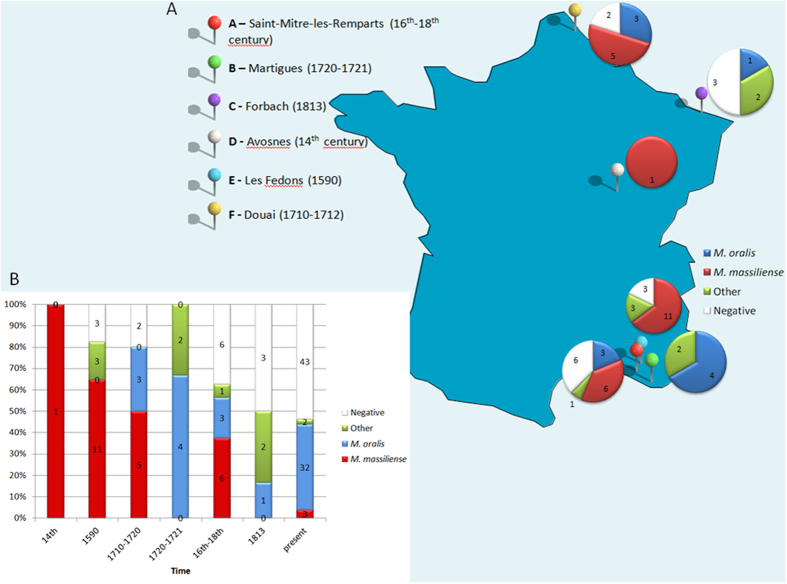
Dental calculus methanogens in France according to geography and time. (**A**) Distribution of dental calculus methanogens detected by PCR-sequencing of the *mcr*A and 16S rRNA genes at six archaeological sites. (**B**) Historical time distribution of methanogens in dental calculus collected from six archeological sites.

A total of 100 dental calculus samples were collected from individual teeth obtained from six archeological sites in France (Fig. 3). These included: 37 samples from the cemetery of Saint-Mitre-les-Remparts (16^th^–18^th^ century) (site A); 11 samples from Martigues, a 1720–1721 plague epidemic burial site (site B); 12 samples from Forbach, a 1813 typhus epidemic site (site C); one sample from Avosnes, a 14^th^ century site (site D); 29 samples from Les Fedons, a 1590 plague epidemic burial site (site E); and 10 samples from Douai, a 1710–1712 typhus site (site F). The majority of studied teeth were separated teeth without bone fragments. It was not possible to assess the sex, age, or periodontopathologic status of the individuals. Each tooth was observed macroscopically and radiographed using digital x-ray cone-beam imaging for 2D and 3D views. Each dental calculus was then collected into a sterile tube using a sterile dental excavator. The repertoires of methanogens in ancient dental calculus of past French populations were compared with that of modern French population recently demonstrated in dental plaque of periodontitis patients and control individuals^11^. The experimenter changed mask, gloves and sterile materials for each new specimen. One sterile tube containing 250 μL of sterile water was left open during the manipulation of ten consecutive specimens and the ten resulting tubes were used as extraction controls. All manipulations were conducted in a room where work on methanogens had never been performed previously. The experiments were conducted under an air hood where no modern specimens were manipulated.

### Molecular detection and identification of methanogens

Each dental calculus was incubated in 125 μL of 0.5 M ethylenediaminetetraacetic acid (EDTA) at pH 8 (Promega, Charbonnières, France), 125 μL of distilled water, 180 μL of T1 solution and 25 μL of proteinase K (Macherey-Nagel, Hoerdt, France) at 56 °C overnight and total DNA was extracted as previously described^3^. Ten μL of a synthetic, internal control plasmid suspension were incorporated into all tubes, including extraction control tubes, to screen for any PCR inhibition using specific primers. PCR sequencing of 16S rRNA and *mcr*A genes was performed as previously described^6^,^19^. Briefly, 5 μL of extracted DNA were amplified in a 25-μL PCR mixture consisting of 2.5 μL of buffer (Qiagen, Courtaboeuf, France), 1 μL of MgCl_2_, 2.5 μL of 2 mM dNTP, 0.5 μL of each primer at a concentration of 10 μM, 0.1 μL of 1X bovine serum albumin and 0.25 μL of Hotstart Taq DNA polymerase (Qiagen). PCR was performed under the following conditions: 95 °C for 15 min and 40 cycles at 95 °C (30 s), 58 °C for 16 S/60 °C for *mcr*A (30 s), and 72 °C (45 s), followed by a 15-min extension at 72 °C. Distilled water was used as negative control in each PCR run. The ten extraction controls were also included in PCR inhibition screening and PCR sequencing of the two methanogen genes. The amplification steps were performed in a separate room from the one in which dental calculus samples were processed and different from the one in which the PCR mix was prepared. The sequences were analyzed with the ChromasPro program, version 1.5 and default parameters and similarity values were determined using the online BLAST program (blast.ncbi.nlm.nih.gov). In the event of ambiguities relating to >50% of the sequence, suggesting multiple amplifications, the PCR-amplified sequence was cloned using Plasmid pGEM^®^-T Easy Vector according to the manufacturer’s instructions (Promega, Charbonnières, France) and the cloning library was screened for appropriately sized inserts. Inserts were sequenced using amplicon-specific primers. Sequences were incorporated into a neighbor-joining phylogenetic tree using the maximum likelihood method with MEGA5. A 98% similarity in the 16S rRNA gene sequence was used as a cut-off to identify methanogens at the species level. The 16S rRNA sequences were deposited in GenBank under accession number LN610761-LN610766 and LN827537-LN827543, while the *mcr*A sequences were deposited under accession numbers LN624393-LN624397.

### Fluorescence *in situ* hybridization

The microbial community structure was blindly studied by fluorescence *in situ* hybridization (FISH) in four PCR-negative dental calculi, six PCR-positive for *Candidatus* Methanobrevibacter sp. N13 and six PCR-positive for *Methanobrevibacter oralis* calculus. FISH incorporated probe EUB338 5′-GCTGCCTCCCGTAGGAGT-3′ labeled with Alexa fluor-546 and specific for Eubacterial 16S rRNA gene and probe ARC915 5′-GTGCTCCCCCGCCAATTCCT-3′ labeled with Alexa fluor-488 and specific for Archaeal 16S rRNA gene. *Escherichia coli* was used as a control. Experiments were conducted as previously described^20^ with modifications. Briefly, calculus samples were fixed for six hours in 4% paraformaldehyde in phosphate buffer saline containing 0.05 M EDTA pH 7.4, 5 mM CaCl_2_, 5 mM NaHCO_3_, then centrifuged to avoid calcite dissolution. *In situ* hybridization was performed in humidity chambers at 46 °C for six hours.

### Statistical analyses

The χ^2^ test was used to assess the statistical significance of differences in the prevalence of methanogens here observed for historical periods with a theoretical prevalence derived from the one previously published for the modern period in France^11^; and the Cramer’V^21^ was mesured to determine the strength of the test χ^2^. We used three scenarios: ancient populations exhibited the same ratio of healthy/unhealthy individuals as modern population (28.2%)^16^ (scenario 1) or only unhealthy individuals (scenario 2) or only healthy individuals (scenario 3) and the prevalence of *M. oralis* in modern healthy population (30%) and in modern unhealthy population (54.5%)^3^.

## Additional Information

**How to cite this article**: Huynh, H. T. T. *et al*. Restricted diversity of dental calculus methanogens over five centuries, France. *Sci. Rep.* **6**, 25775; doi: 10.1038/srep25775 (2016).

## Figures and Tables

**Figure 1:**
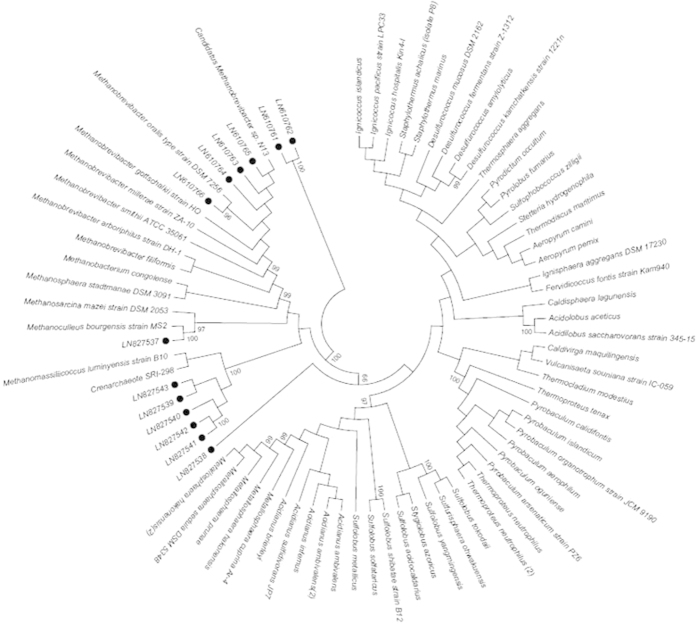
A 16S rRNA gene sequence-based tree indicating the position of four methanogens detected by PCR-sequencing in ancient dental calculus samples in France. Bootrap values ≥95% are indicated at nodes. The 16S rRNA archaeal sequences with their accession number were presented with a bubble.

**Figure 2:**
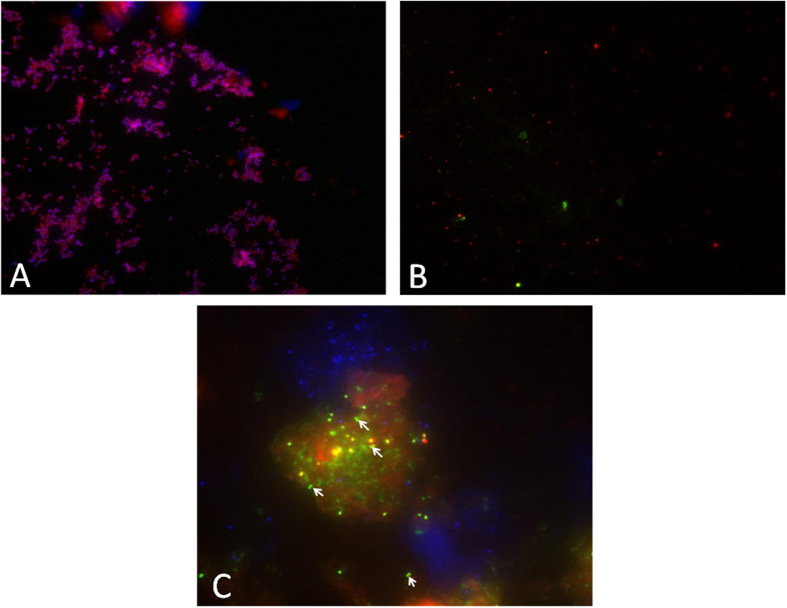
FISH detection of *Candidatus Methanobrevibacter* sp. N13 in one 18^th^ century dental calculus, Douai site, France. (**A**) *E. coli* experimental control of FISH (**A**,**B**) one archaea-negative ancient dental calculus used as negative control and (**C**) one archaea-positive ancient dental calculus specimen. Blue represents DAPI fluorescence staining all microorganisms. Red represents EUB338 fluorescence staining the Bacterial domain. Green represents ARC915 fluorescence staining the Archaeal domain. Arrows show the archaeal evidence under fluorescent microscope.

**Table 1:** Samples used in this study and their identification.

Site	Name of site	Period	Dental calculus samples	Not inhibited samples	*mcr*A (+)	16S (+)	Positive (either 16S+ or *mcr*A+)	Identification			
								*M. oralis*	*Candidatus*Methanobrevibacter sp. N13	*M. bourgensis*	?
A	Saint-Mitre-les-Remparts	16^th^–18^th^ century	37	16	7	8	10	3	6		1
B	Rayettes (Martigues)	1720–1721	11	6	3	6	6	4	0		2
C	Forbach	1813	12	6	3	3	3	1	0	1	1
D	Avosnes	14^th^ century	1	1	1	1	1	0	1		0
E	Les Fedons	1590	29	17	10	14	14	0	11		3
F	Douai	1710–1712	10	10	6	8	8	3	5		0
	Total	14^th^–19^th^ century	100	56	28	40	42	11	23	1	7
